# Role of multifaceted regulators in cancer glucose metabolism and their clinical significance

**DOI:** 10.18632/oncotarget.7765

**Published:** 2016-02-26

**Authors:** Luqing Zhao, Yitao Mao, Yuelong Zhao, Ya Cao, Xiang Chen

**Affiliations:** ^1^ Department of Dermatology, Xiangya Hospital, Central South University, Changsha, Hunan, China; ^2^ Hunan Key Laboratory of Skin Cancer and Psoriasis, Xiangya Hospital, Central South University, Changsha, Hunan, China; ^3^ Department of Pathology, School of Basic Medical Science, Xiangya School of Medicine, Central South University, Changsha, Hunan, China; ^4^ Department of Pathology, Xiangya Hospital, Central South University, Changsha, Hunan, China; ^5^ Department of Radiology, Xiangya Hospital, Central South University, Changsha, Hunan, China; ^6^ Cancer Research Institute, School of Basic Medical Science, Xiangya School of Medicine, Central South University, Changsha, Hunan, China; ^7^ School of Information Sceince and Engineering, Central South University, Changsha, Hunan, China

**Keywords:** glucose metabolism, multifaceted regulators, p53, HIF-1, TIGAR

## Abstract

Aberrant glucose metabolism, “aerobic glycolysis” or “Warburg effect”, is a hallmark of human cancers. There is a cluster of “*multifaceted regulators*”, which plays a pivotal role in the regulation of glucose metabolism. They can not only modulate the activities of specific enzymes, but also act as transcriptional activators to regulate the expression of metabolism related genes. Additionally, they can crosstalk with other key factors involved in glucose metabolism and work together to initiate multiple oncogenic processes. These “*multifaceted regulators*”, especially p53, HIF-1, TIGAR and microRNA, will be focused in this review. And we will comprehensively illustrate their regulatory effects on cancer glucose metabolism, and further elaborate on their clinical significance. In-depth elucidation the role of “*multifaceted regulators*” in cancer glucose metabolism will provide us novel insights in cancer research field and offer promising therapeutic targets for anti-cancer therapies.

## INTRODUCTION

Reprogramming of cancer metabolism is a well established hallmark of the transformed phenotype, which demands a rather high rate of glycolysis in order to satisfy the increasing requirements for macromolecular synthesis (such as nucleotides, lipid and protein) and maintain rapid proliferation [1]. This metabolic state is quite different from that of normal cells. Tumor cells take up much more glucose and rely on glycolysis, even in the presence of abundant oxygen [2]. Indeed, even though the TCA cycle and mitochondrial OXPHOS would generate more ATP, cancer cells choose to utilize less efficient glycolysis producing large quantities of pyruvate and lactate. This phenomenon is known as “aerobic glycolysis” or the “Warburg effect”, which is a usual event in multiple cancers [3, 4]. Exploiting this metabolic reprogramming, cancer cells utilize the low energy *via* glycolysis to fuel the malignant phenotype [5]. They are much more apt to survive under hypoxic stress conditions and become more resistant to cell death, at the same time, promoting cell proliferation and metastasis [6]. The “addiction” of cancer cells to glucose metabolism is confirmed by ^18^fluorodeoxyglucose (^18^FDG) positron-emission tomography (PET), which has shown that many cancers have higher uptake of glucose relative to normal tissues [7].

The metabolic reprogramming in cancer cells also alters the levels of other intermediates and substrates implicated in glycolysis [8]. For instance, increased glycolysis can generate more NADPH by other pathways like the pentose phosphate pathway (PPP), and decrease the levels of reactive oxygen species (ROS) so as to protect the cell from oxidative stress [9]. Moreover, the pyruvate made during glycolysis is commonly converted to lactate, most of which is secreted from cancer cells. The secreted lactate will lower the pH value of the extracellular matrix (ECM) [10]. An acidic tumor microenvironment will facilitate the motility of cancer cells, and these cells can break through the basement membrane and metastasize [11]. In addition, the acidosis of the tumor microenvironment will increase the resistance of cancer cells to radiation or chemotherapy [12].

It is widely acknowledged that numerous factors are involved in the regulation of cancer glucose metabolic reprogramming. Oncogenes, tumor suppressor genes and transcriptional activators modulate the glycolytic pathway in an orderly manner [13]. Among them, the PI3K/AKT pathway plays a pivotal role and influences multiple processes in glucose metabolism; c-Myc promotes the efficiency of glycolysis [14]; NF-κB, FOXO3A, STAT3, PKM2 also act as transcriptional factors that affect the expression of metabolism related genes [15–18]. Meanwhile, there is also a cluster of “*multifaceted regulators*”, such as p53, HIF-1, TIGAR and microRNA, which perform multiple roles in different genetic settings and influence various aspects of malignant progressions, including proliferation, migration, metastasis, angiogenesis, metabolic reprogramming and chemo- or radio-resistance.

In this review, we mainly focus on the role of four multifaceted regulators in cancer glucose metabolic reprogramming, illustrating their potential regulatory mechanisms in detail, and finally highlighting their clinical significance for exploring new therapeutic targets.

## KEY MULTIFACETED REGULATORS INVOLVED IN CANCER GLUCOSE METABOLISM

### p53 and cancer glucose metabolism

p53, a classic tumor suppressor gene, is a well-studied versatile transcription factor involved in a wide range of cellular processes including genome integrity maintenance, cell survival, angiogenesis, stemness, metabolism, epithelial-mesenchymal transition, fertility, aging, autophagy and especially the control of cell cycle progression and apoptosis [19–22]. p53 has also emerged as a key player in the DNA damage response pathway, which can be activated by DNA-damaging agents, resulting either in triggering cell cycle checkpoint to promote cell survival or in inducing apoptosis [23]. p53 can interplay with various oncogenes and/or tumor suppressors, such as c-Myc, NF-κB and HIF-1, and may affect the activity of transcriptional factor in nucleus or mitochondria [24]. Additionally, p53 could negatively regulate the PI3K/AKT/mTOR pathway through targeting IGF-BP3, PTEN, Sestrin1/2, TSC2, AMPK subunit, and so on. And all of these p53 targeted genes play an essential role in response to metabolic stresses [25]. Also, p53 can crosstalk with numerous endogenously expressed microRNAs so as to form a complex p53-microRNA network and regulate the transcription, expression and maturation of a group of functionally essential microRNAs [26].

**Figure 1:**
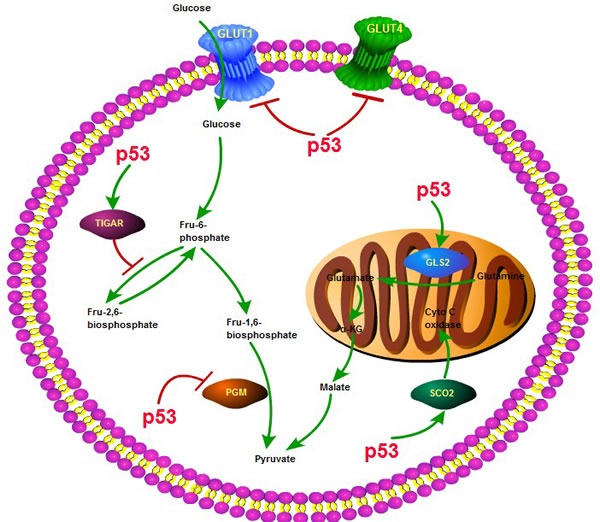
The role of p53 in glycolysis and oxidative phosphorylation p53 plays a key role in the process of glycolysis and oxidative phosphorylation, through interacting with various molecules or enzymes, such as SCO2, TIGAR, GLUT1,4, GLS2, PGM and affecting several key biological processes including glucose uptake, glutamine generation and pyruvate conversion.

In addition to its crucial involvement in numerous biological functions, multifaceted p53 plays a central role in aerobic glycolysis and other aspects of glucose metabolism reprogramming [27–30]. Besides directly impairing the activities of metabolic enzymes, p53 can act as a transcriptional factor to modulate the transcription of various metabolism related genes [31]. In the glycolysis process, p53 represses the transcription of glucose transporter 1 (GLUT1) and GLUT4, so as to reduce glucose uptake from the tumor microenvironment [32]. p53 also induces the transcription of TIGAR (TP53-induced glycolysis and apoptosis regulator), whose expression will lower the levels of fructose-2,6-bisphosphate and intracellular reactive oxygen species (ROS) [33]. Moreover, the activation of p53 may increase the ubiquitination of phosphoglycerate mutase (PGM) and prevent fructose-1, 6-bisphosphate changing into pyruvate. In the OXPHOS process, p53 not only increases the use of TCA cycle, but also elevates the transcription of synthesis of cytochrome c oxidase 2 (SCO2), which is required for assembly of the cytochrome c oxidase subunit 4 (COX4) and insurance of its maintenance,so as to promote the entry into the mitochondrial oxidative respiratory chain [34]. Besides, p53 can affect the transcription of glutaminase 2 (GLS2), which enhances the activity of TCA cycle and upregulates the rate of OXPHOS [35]. To conclude, the multifaceted role of p53 in cancer glucose metabolism is manifested in inhibiting the glycolysis process and facilitating the TCA cycle and OXPHOS. In the absence of p53, tumorigenesis will be increased, at least in part because the rate of glucose metabolic reprogramming would be greatly accelerated (Figure 1).

### HIF-1 and cancer glucose metabolism

Hypoxia-inducible factor 1 (HIF-1) is recognized as a master regulator of the transcriptional response to hypoxia, which is a low oxygen level frequently detected in tumor microenvironment [36]. HIF-1 is a critical transcription factor in various cellular and physiologic processes, as it can facilitate adaption of tumor cells to hypoxia by activating the transcription of downstream target genes and regulating multiple aspects of tumorigenesis, including cell proliferation, survival, differentiation, apoptosis, angiogenesis, immunosurveillance, metabolism, metastasis, as well as radiation response [37–39]. Overexpression of HIF-1 has been associated with resistance to radio- or chemotherapy, increased risk of invasion and migration, and a poor clinical outcome in patients with solid tumors [40]. HIF-1 induction is associated with a multitude of downstream effects, including angiogenesis, achieved by increasing the expression of a number of angiogenic growth factors that are under direct HIF-1 transcriptional control [41]. Blocking the expression of HIF-1 can effectively prevent the progression of angiogenesis and profoundly change the tumor microenvironment [42].

HIF-1 also plays a multifaceted role in regulating cancer glucose metabolism reprogramming [43–45]. HIF-1 can regulate glucose metabolism at different levels and has a profound effect on glycolysis and the pentose phosphate pathway [46]. When HIF-1 is activated, the efficiency of the glycolytic pathway is increased, and the mitochondrial OXPHOS is suppressed [47]. HIF-1 directly regulates the expression and function of several key metabolic enzymes involved in glycolysis. More specifically, glucose transporters GLUT1 and GLUT3, which promote the glucose entry into tumor cells, are main targets of HIF-1, and can effectively increase the availability of glucose [48]. Hexokinase 2 (HK2) is another major target of HIF-1, which enhances the phosphorylation of glucose [49]. Phosphoglucose isomerase (PGI), phosphofructokinase 1 (PFK1), triosephosphate isomerase (TPI), glyceraldehyde-3-phosphate dehydrogenase (GAPDH), phosphoglycerate kinase (PGK), phosphoglycerate mutase (PGM) and pyruvate kinase (PK) are a cluster of HIF-1 targets which play essential roles in promoting glycolysis [50]. Other target genes, like lactate dehydrogenase A (LDHA) and monocarboxylate transporter 4 (MCT4), can facilitate both the conversion of pyruvate to lactate and the removal of lactate from tumor cells [51]. Additionally, pyruvate dehydrogenase kinase 1 (PDK1), max interactor 1 (MXI1) and COX4 are also under the control of HIF-1 and contribute to repressing mitochondrial activities and decreasing oxygen consumption in hypoxia [52].

**Figure 2:**
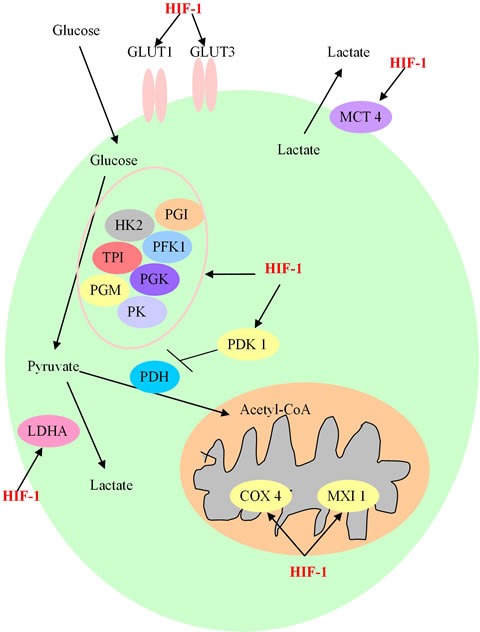
The involved procedure of HIF-1 in cancer glucose metabolism HIF-1 enhances the expression of glucose transporters GLUT1 and GLUT3, and activates glycolytic enzymes, including Hexokinase 2 (HK2), Phosphoglucose isomerase (PGI), phosphofructokinase 1 (PFK1), triosephosphate isomerase (TPI), phosphoglycerate kinase (PGK), phosphoglycerate mutase (PGM) and pyruvate kinase (PK) to generate increasing amount of pyruvate. After this process, pyruvate is largely converted to lactate by lactate dehydrogenase A (LDHA) and removed out from cancer cell by monocarboxylate transporter 4 (MCT4). HIF-1 also prevents the TCA cycle and oxidative phosphorylation process by activating the expression of HIF-1-dependent pyruvate dehydrogenase kinase 1 (PDK1), max interactor 1 (MXI1) and cytochrome c oxidase subunit 4 (COX4), resulting in the decrease of mitochondrial activities and the oxygen consumption in hypoxia.

Overall, the role of HIF-1 in cancer glucose metabolic reprogramming can be summarized as follows: (1) Through the up-regulation of glucose transporters GLUT1 and GLUT3, there is an increased uptake of glucose into tumor cells [53]; (2) The intracellular glucose is metabolized by the activated glycolytic enzymes to enter the glycolytic pathway rather than the OXPHOS process; (3) With the increased ongoing glycolysis, a large amount of pyruvate is generated, most of which is then converted into lactate by LDHA and excreted by MCT, rather than flowing into the TCA cycle in mitochondria [54]; (4) The HIF-1-dependent induction of PDK1 will decrease the flow through the TCA cycle, and the induction of MXI1 in mitochondria will suppress the biogenesis of mitochondria and decrease the process of OXPHOS [55, 56]. In conclusion, HIF-1 may be a potential therapeutic target to inhibit tumor metabolism by affecting multiple steps implicated in this process (Figure 2).

### TIGAR and cancer glucose metabolism

TP53-induced glycolysis and apoptosis regulator (TIGAR) is a downstream target gene of p53 and a regulator of glycolysis and apoptosis, whose expression level can be transactivated by p53 [57]. TIGAR is involved in various biological processes, including metabolism, apoptosis, cell cycle, cell death, autophagy, migration, metastasis, and radiation response [58]. It has been reported that the protein levels of TIGAR can be increased shortly after irradiation, which raises the possibility of considering TIGAR as a new therapeutic target to increase radiotherapy effects and to allow lower radiation doses to achieve the same therapeutic effect [59]. Overexpression of TIGAR can reduce cell death, as it is induced by the restriction of glucose and oxygen supply [60].

When it comes to the glucose metabolism field, metabolic analyses have revealed that TIGAR could inhibit glycolysis and promote oxidative respiration. By regulating the transcriptional activity of TIGAR, p53 can efficiently modulate the aerobic respiration at multiple glycolytic and OXPHOS steps [61]. TIGAR has a bisphosphatase domain similar to that of 6-phosphofructo-2-kinase (PFK-2) and fructose-2, 6-bisphosphatase (F26BPase), which are both indispensable for the glycolytic process [62]. TIGAR, acting as an inhibitor of the fructose-2, 6-bisphosphate (F26BP), will lower the intracellular F26BP level and result in a decreased glycolytic rate [63]. Owing to the activation of TIGAR, p53 can negatively regulate glycolysis. Knockdown of p53 or TIGAR will increase glycolysis rate with the elevation of F26BP level and the reduction of apoptosis process. In contrast, overexpression of TIGAR will reduce the utilization of glucose and increase apoptosis related processes [64].

Moreover, hypoxia can induce the expression of TIGAR in a p53-dependent manner and TIGAR can inhibit glycolysis in a hypoxic microenvironment. Meanwhile, the inhibition of glycolysis is closely involved in the regulation of apoptosis. Therefore, induction of TIGAR expression is a crucial mediator of cellular energy homeostasis under hypoxic stress [65]. Also, in the presence of hypoxia, TIGAR can relocalize to mitochondria and form a complex with HK2, leading to an increase in HK2 activity. This mitochondrial localization of TIGAR is largely dependent on the mitochondrial HK2 and HIF-1 activity. The final effect of this mitochondrial interaction between TIGAR and HK2 may be the limitation of mitochondrial ROS production and protection of cells from death [66]. Another major function of TIGAR in cancer metabolism is to increase the level of NADPH and lower the intracellular level of ROS [67]. Through the pentose phosphate pathway, the increased NADPH production can help to limit the ROS level. However, ROS take an important role in the regulation of cellular responses, such as the responses to nutrient starvation or metabolic stresses [68].

Meanwhile, the intracellular ROS levels are closely correlated with autophagy, oxidative stress-associated apoptosis and a higher resistance to cell death [69]. The expression of TIGAR can not only protect cells from ROS-associated apoptosis and the accumulation of genomic damage, but also act as a role similar to p53, to modulate apoptotic responses [70]. So TIGAR functions to regulate the glycolytic pathway and lower the ROS levels, in order to protect cells from oxidative stress [71]. In short, TIGAR is essential in cancer glucose metabolism, and has the potential to be a novel therapeutic target for cancer therapy. Targeting metabolic regulators like TIGAR may become a valuable approach to enhance ROS related therapeutic strategies and prevent multiple processes in glucose metabolism.

### MicroRNA and cancer glucose metabolism

MicroRNA is a class of small non-coding RNA, of about 22 nucleotides in length, which can bind to the 3′;-untranslated region (3′;-UTR) of target mRNA and thereby inhibit mRNA translation or promote mRNA degradation at the post-transcriptional levels [72]. Accumulating evidence has demonstrated that microRNA is correlated with multiple aspects of cancer biology, including control of cell growth, proliferation, differentiation, cell cycle, apoptosis, cell death, migration and metastasis [73, 74]. In recent years, the role of microRNA as a key regulator of cancer metabolism has drawn increasing attention. MicroRNA could regulate cancer metabolic processes and facilitate the “Warburg effect” through targeting key metabolic enzymes or glucose transporters, regulating the activities of metabolism related transcription factors, oncogenes/tumor suppressors as well as interplaying with various oncogenic signaling pathways [75, 76].

The pivotal regulatory role of microRNA in cancer metabolism nearly covers every aspect of metabolic reprogramming, including glucose uptake, glycolysis, TCA cycle, glutamine production, amino acid biogenesis as well as lipid metabolism [77]. In the context of cancer glucose metabolism, microRNA serves as a multifaceted master, which can modulate the expression of glycolysis related genes either by directly activating metabolic machinery or indirectly regulating the activities of metabolic enzymes [78]. It has been reported that miR-143 could regulate glycolysis in cancer cells by targeting the first rate-limiting enzyme hexokinase 2 (HK2). MiR-143 expression level inversely correlates with HK2 protein levels. By targeting HK2, miR-143 reduces glucose metabolism and inhibits cancer cell proliferation. As a crucial regulator of cancer glycolysis, miR-143 offers potential clinical therapeutic prospects [79].

The expression of miR-155, induced by pro-inflammatory cytokines, can also regulate glucose metabolism and promote glycolysis [80]. MiR-155 activates the expression of signal transducer and activator of transcription 3 (STAT3), which is a transcriptional activator of HK2 and up-regulates its expression level. Besides, by targeting a transcriptional activator (C/EBPβ) of miR-143, miR-155 could inhibit the expression of miR-143, which is a negative regulator of HK2, therefore leading to the up-regulation of HK2 expression at the post-transcriptional level [81]. These findings indicate that by modulating the miR-143/HK2 axis, miR-155 not only controls the aerobic glycolysis (or Warburg effect) in cancer cells, but also provides a possible mechanism to explain the link between inflammation and the altered glucose metabolism in cancer. Additionally, miR-195-5p has been shown to directly target the 3′;-UTR of GLUT3 and regulate its expression in order to suppress glucose uptake, inhibit tumor cell growth and proliferation, and promote apoptosis of tumor cells. Therefore, the decreased expression of miR-195-5p will lead to the up-regulation of GLUT3 expression, which may contribute to tumorigenesis [82].

**Figure 3:**
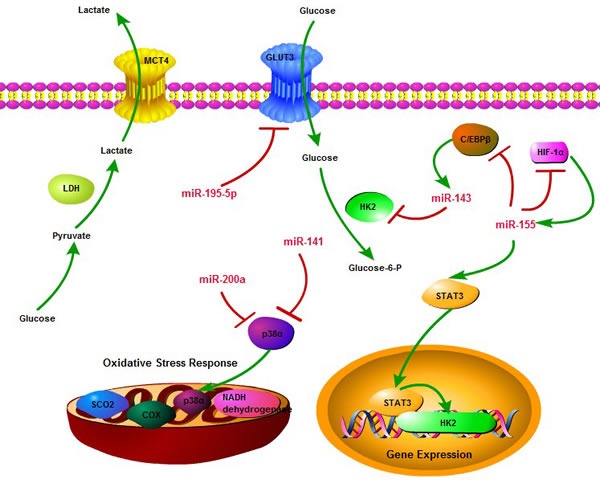
The regulatory network of microRNA in cancer glucose metabolism microRNA can regulate cancer glucose metabolism through diverse aspects, including glucose uptake, glycolysis, oxidative phosphorylation, and multiple targets, including GLUT3, HK2, HIF-1α, p38α, STAT3, so as to consist of a complicated network to influence essential links in cancer metabolism. Meanwhile, miR-143 and miR-155 form a negative feedback loop to control glycolysis process.

Meanwhile, miR-141 and miR-200a may control tumor oxidative stress response and regulate redox potential by targeting p38α. Over-expression of these microRNAs contributes to p38α deficiency and the enhanced chemosensitivity. This study reveals that miR-200a or p38α could become a potential oxidative stress signature, predictive of clinical outcome and treatment response to chemotherapeutic agents in cancer patients [83]. Besides, during prolonged hypoxia, miR-155 may target HIF-1α mRNA and decrease HIF-1α activity through an isoform-specific negative-feedback loop. Furthermore, HIF-1α could elevate the expression of miR-155 by interacting with the hypoxia response elements in its promoter regions. This microRNA-HIF-1α interaction loop provides new insights in the regulation of HIF-1α-dependent transcriptional activities and its downstream biological effects, including cancer cell metabolism [84]. In conclusion, the effects of microRNA deregulation in metabolic processes may lead to the progressive conversion of normal cells into cancer cells with increased malignant properties. Finally, the critical role that microRNA play in cancer glucose metabolic reprogramming may provide a novel therapeutic approach for the treatment of highly metabolic cancers and promote the development of microRNA-based strategies for cancer therapy (Figure 3).

### The crosstalk between microRNA and other multifaceted regulators in cancer glucose metabolism

As a key regulator of post-transcriptional gene expression, microRNA has crosstalk with above multifaceted regulators in cancer glucose metabolism and interplays with them, so as to make the glucose metabolism regulatory system more complicated. The final effect of microRNA in determining the metabolism reprogramming is mainly through the interaction with oncogene (eg: HIF-1) and tumor suppressor (eg: p53, TIGAR) networks which directly influence the metabolic switch in cancer. More specifically, miR-34a is a key downstream target of p53, and p53-inducible miR-34a can suppress the expressions of multiple glycolytic enzymes, including hexokinase 1 (HK1), HK2, glucose-6-phosphate isomerase (GPI), and PDK1, which result in repressed glycolysis process and enhanced mitochondrial respiration. It is indicated that p53 has a miR-34a-dependent integrated mechanism to regulate glucose metabolism. In cancer cells, p53-miR-34a network can lead to the inhibition of Warburg effect and the promotion of OXPHOS procedure to utilize glucose [85]. Furthermore, miR-34a can directly target sirtuin 1 (SIRT1), which is an essential modulator in cellular metabolism. SIRT1 deacetylates and activates the transcriptional activities of metabolic regulators, such as PGC-1α, p53, FoxO1, NF-κB, LXR, and FXR which are involved in glucose metabolism, mitochondrial biogenesis, and energy balance control. Meanwhile, SIRT1 positively auto-regulates its own expression by inhibiting miR-34a *via* deacetylation of p53 and the histones at the miR-34a promoter region and further can be recruited to the promoters of metabolic target genes and regulate their transcription [86]. In addition, in high glucose condition, p53 mediates the inhibition of miR-17-92, which is a repressor of Bim, so as to permit the accumulation of Bim and promote cell apoptosis. High glucose coupled with oxidative stress results in the upregulation of miR-28-5p, which directly inhibits the expression of p53 deacetylase SIRT3 and leads to the increased level of acetylated p53 [87].

**Figure 4:**
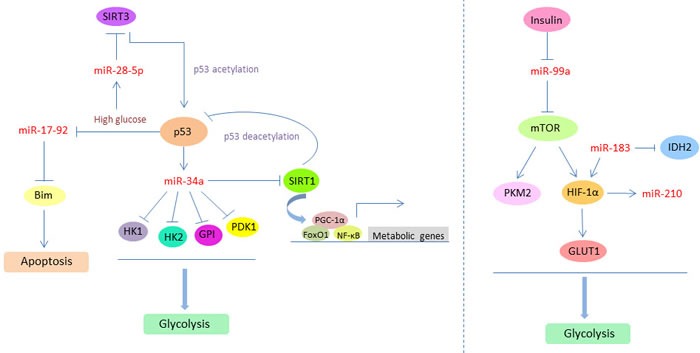
The crosstalk between microRNA and multifaceted regulators (p53 and HIF-1) in cancer glucose metabolism p53 and HIF-1 can induce the expression of specific microRNAs, such as miR-34a and miR-210, and further mediate the biological functions of microRNA targets which are involved in glycolysis process. Meanwhile, the expression levels or activities of p53 and HIF-1 are also under the direct or indirect control of several microRNAs, such as miR-183, miR-28-5p, and miR-99a, through the acetylation and deacetylation modification. The feedback loop and crosstalk between microRNAs and these two essential metabolic regulators can effectively modulate cancer glucose metabolism and promote the Warburg effect.

The crosstalk between microRNA and HIF-1 in cancer glucose metabolism seems more complex. Insulin can inhibit the expression of miR-99a, and then induce the expression of miR-99a direct target mTOR, which in turn increases PKM2 and HIF-1α expression for regulating glucose consumption and lactate production. Knockdown of HIF-1α inhibits PKM2 expression and insulin-induced glucose consumption. These findings reveal the role of insulin in regulating glycolytic activities *via* miR-99a/mTOR/HIF-1α pathway and indicate the intimate relationship between cancer glucose metabolism and diabetes [88]. Moreover, miR-183 directly targets isocitrate dehydrogenase 2 (IDH2) and down-regulates its expression in glioblastomas. Overexpression of miR-183 or inhibition of IDH2 can contribute to the up-regulation of HIF-1α and its downstream molecule GLUT1 so as to facilitate glucose uptake and glycolysis process in tumor cells [89]. Additionally, miR-210 is a unique HIF-responsive “hypoxamir” that is evolutionarily conserved and ubiquitously expressed in hypoxic cells and tissues. miR-210 disrupts mitochondrial respiration and inhibits glucose metabolism *via* TCA cycle, resulting in a metabolic shift from mitochondrial OXPHOS to glycolysis and accelerating the Warburg effect of cancer cells [90]. In sum, the crosstalk between microRNA and multifaceted regulators, especially the p53 and HIF-1, in cancer glucose metabolism, will help us better understanding the whole picture of these key metabolic modulators mediated regulatory networks and the central role that microRNA played in controlling glucose metabolism. It will also provide new aspects to explore promising therapeutic targets to reverse or eradicate cancer glucose metabolism abnormality (Figure 4).

## CLINICAL SIGNIFICANCE OF GLUCOSE METABOLISM TARGETS

There are multiple therapeutic targets in the glucose metabolic pathway, which could be potential targets in anti-cancer strategies and offer promising clinical potential. Specifically, the approaches can be divided into four parts, which aim at inhibiting glycolytic and pentose phosphate pathway enzymes, promoting the OXPHOS process and attenuating the activity of HIF-1 [91]. In the field of targeting glycolytic enzymes, there are several agents in preclinical development or approaching clinical trials, including hexokinase targeted drugs (Lonidamine, 2-Deoxyglucose and 3-Bromopyruvate), pyruvate kinase targeted drugs (TLN-232) and 6-Phosphofructo-1-kinase targeted drugs (3PO). All of these agents can effectively suppress, albeit to a different extent, the activity of glycolytic enzymes [92, 93]. Moreover, novel drugs like Oxythiamine or 6-Aminonicotinamide could directly target crucial enzymes (Transketolase and Glucose-6-dehydrogenase) in the pentose phosphate pathway so as to limit the glucose use by this route [94]. Another strategy to control cancer glucose metabolism is the promotion of OXPHOS. Examples in this regard have been provided by using the RNA interference technique to repress the expression of lactate dehydrogenase, or Dichloroacetate to target the pyruvate dehydrogenase kinase, approaches that may have potential anti-cancer effects [95, 96].

In addition, blocking HIF-1 activity can be an effective approach to alter glucose metabolism in cancer cells. Several mechanisms of HIF-1 inhibition have been reported: agents like Topotecan, Digoxin or PX-478, may inhibit the translation or protein synthesis of HIF-1α; Topotecan which is approved by FDA to treat several cancers, including ovarian cancer and small-cell lung cancer, has also been tested in clinical trials to target HIF-1 [97]. YC-1 and Echinomycin, could selectively affect the stability of HIF-1 or its DNA binding activity. There are numerous other potential therapeutic targets in the metabolism-related signal transduction pathways, including the PI3K/AKT/mTOR pathway and AMPK pathway. More specifically, GDC-0941 and Perifosine can directly inhibit the function of PI3K and AKT; Temsirolimus can directly suppress the expression of mTORC1; BEZ235 can effectively block the PI3K/mTOR signaling; Metformin can otherwise activate AMPK expression and affect the glucose and insulin metabolism [98, 99]. Many of these drugs are being tested in clinical trials in patients with advanced solid tumors or lymphomas [100–102]. Further exploring the potential clinical applications of strategies targeting glucose metabolism will bring us novel insights and avenues for the development of promising anti-cancer therapies.

## CONCLUDING REMARKS

Cancer glucose metabolism is a novel, emerging hallmark of cancer cells, which is represented by the “aerobic glycolysis” or “Warburg effect”, and aims to increase the synthesis of macromolecules and intermediates to maintain tumor growth and proliferation. In recent years, the study of glucose metabolism in cancer cells has become a rapidly growing area of research. Mounting evidence shows that numerous multifaceted factors are involved in this process. Among them we have highlighted the tumor suppressor gene p53, the transcription factor HIF-1α, TIGAR, and specific microRNAs (Table 1). These molecules, by interacting with crucial transcription factors or metabolic enzymes involved in the processes of glycolysis and OXPHOS, can efficiently modulate glucose metabolism and enhance tumor cells survival. Besides these critical molecules, additional metabolic-related pathways are also crucial for cancer glucose metabolism, especially the PI3K/AKT/mTOR pathway and the AMPK pathway, in particular when tumor cells are exposed to growth factors.

**Table 1:** Role of key multifaceted regulators in cancer glucose metabolism

Metabolism procedures	Key molecules	Potential targets	Biological effects	Refs
Glycolysis process	p53	GLUT1 and GLUT4	Prevent the glucose uptake	[32]
	HIF-1	GLUT1 and GLUT3	Promote the glucose entry into the tumor cells	[48]
	HIF-1	HK2	Enhance the phosphorylation of glucose	[49]
	HIF-1	LDHA and MCT4	Facilitate the conversion of pyruvate to lactate and the removal of lactate from tumor cells	[51]
	TIGAR	Fructose-2, 6-bisphosphate	Decrease the glycolytic rate	[63]
	TIGAR	NADPH	Lower the intracellular ROS level	[67]
	miR-143	HK2	Reduce glucose metabolism and inhibit tumor cell proliferation	[79]
	miR-155	STAT3 and C/EBPβ	Control the aerobic glycolysis of tumor cells	[81]
	miR-195-5p	GLUT3	Suppress the glucose uptake, inhibit tumor cell growth and promote apoptosis of tumor cells	[82]
Oxidative phosphorylation process	p53	SCO2 and GLS2	Increase the use of TCA cycle and up-regulate the rate of oxidative phosphorylation	[34, 35]
	HIF-1	PDK1, MXI1 and COX4	Repress mitochondrial activities and decrease oxygen consumption in hypoxia	[52]
	miR-141, miR-200a	p38α	Control tumor oxidative stress response and regulate redox potential	[83]

Abbreviation: GLUT1: Glucose transporter 1; SCO2: Synthesis of cytochrome c oxidase 2; GLS2: Glutaminase 2; TCA cycle: Tricarboxylic acid cycle; HIF-1: Hypoxia-inducible factor 1; HK2: Hexokinase 2; LDHA: Lactate dehydrogenase A; MCT4: Monocarboxylate transporter 4; PDK1: Pyruvate dehydrogenase kinase 1; MXI1: Max interactor 1; COX4: Cytochrome c oxidase subunit 4.

Notably, the metabolic switch that characterizes cancer cells may provide novel attractive targets for cancer therapy. There is growing evidence that supports the potential role of many glycolytic enzymes, transporters or transcription factors as promising candidate targets for cancer treatment. Therefore, thoroughly exploring the regulatory mechanisms of these versatile molecules and their clinical significance in cancer metabolism will help to identify novel ways to control the aberrant metabolic phenotype and find more effective therapeutic strategies to suppress the “Warburg effect” and restore the normal OXPHOS in tumor cells, ultimately paving the way for better prevention and treatment of cancer.
